# Association of Fidaxomicin with *C*. *difficile* Spores: Effects of Persistence on Subsequent Spore Recovery, Outgrowth and Toxin Production

**DOI:** 10.1371/journal.pone.0161200

**Published:** 2016-08-24

**Authors:** Caroline H. Chilton, Grace S. Crowther, Helen Ashwin, Chris M. Longshaw, Mark H. Wilcox

**Affiliations:** 1 Leeds Institute for Biomedical and Clinical Sciences, University of Leeds, Leeds, United Kingdom; 2 Faculty of Science and Engineering, Manchester Metropolitan University, Manchester, United Kingdom; 3 Astellas Pharma Europe Ltd, Chertsey, Surrey, United Kingdom; 4 Department of Microbiology, Leeds Teaching Hospitals NHS Trust, The General Infirmary, Old Medical School, Leeds, United Kingdom; Universidad Andres Bello, CHILE

## Abstract

**Background:**

We have previously shown that fidaxomicin instillation prevents spore recovery in an in-vitro gut model, whereas vancomycin does not. The reasons for this are unclear. Here, we have investigated persistence of fidaxomicin and vancomycin on *C*. *difficile* spores, and examined post-antibiotic exposure spore recovery, outgrowth and toxin production.

**Methods:**

Prevalent UK *C*. *difficile* ribotypes (n = 10) were incubated with 200mg/L fidaxomicin, vancomycin or a non-antimicrobial containing control for 1 h in faecal filtrate or Phosphate Buffered Saline. Spores were washed three times with faecal filtrate or phosphate buffered saline, and residual spore-associated antimicrobial activity was determined by bioassay. For three ribotypes (027, 078, 015), antimicrobial-exposed, faecal filtrate-washed spores and controls were inoculated into broth. Viable vegetative and spore counts were enumerated on CCEYL agar. Percentage phase bright spores, phase dark spores and vegetative cells were enumerated by phase contrast microscopy at 0, 3, 6, 24 and 48 h post-inoculation. Toxin levels (24 and 48h) were determined by cell cytotoxicity assay.

**Results:**

Fidaxomicin, but not vancomycin persisted on spores of all ribotypes following washing in saline (mean = 10.1mg/L; range = 4.0-14mg/L) and faecal filtrate (mean = 17.4mg/L; 8.4–22.1mg/L). Outgrowth and proliferation rates of vancomycin-exposed spores were similar to controls, whereas fidaxomicin-exposed spores showed no vegetative cell growth after 24 and 48 h. At 48h, toxin levels averaged 3.7 and 3.3 relative units (RU) in control and vancomycin-exposed samples, respectively, but were undetectable in fidaxomicin-exposed samples.

**Conclusion:**

Fidaxomicin persists on *C*. *difficile* spores, whereas vancomycin does not. This persistence prevents subsequent growth and toxin production in vitro. This may have implications on spore viability, thereby impacting CDI recurrence and transmission rates.

## Introduction

*Clostridium difficile* infection (CDI) continues to be a leading infective cause of antibiotic-associated diarrhoea, placing substantial burdens on healthcare systems worldwide.[1, 2] Morbidity and mortality, particularly associated with recurrent disease, remain problematic, with ~25% of patients experiencing a recurrence of symptoms following treatment.[3, 4] A key contributing factor to the high rates of recurrence associated with CDI is the persistence of *C*. *difficile* spores in the intestinal lumen (or mucosal associated biofilm) following treatment.[5] These are unaffected by standard antimicrobial therapy, and provide a key reservoir for recurrent disease, allowing re-establishment of a vegetative *C*. *difficile* population following successful initial treatment.[6, 7]

For many years, treatment options were limited to vancomycin and metronidazole. However, the recent introduction of fidaxomicin offers a therapeutic alternative. In phase III clinical trials fidaxomicin was non inferior to vancomycin for initial clinical cure, but was superior in preventing recurrence and sustained clinical sure.[8–10]

We have previously observed that fidaxomicin activity continues to be detected for prolonged periods of time in an *in vitro* gut model,[7, 11] whereas vancomycin activity quickly washes out.[12] Similar prolonged detection of antimicrobial activity has been observed for both ramoplanin [13] and oritavancin,[14] and has correlated with an association of antimicrobial activity on *C*. *difficile* spores, which persists despite washing.[15, 16] We have termed this ‘persistence of activity’. In this study, we have quantified the persistence of fidaxomicin activity on *C*. *difficile* spores, and demonstrate that this persistence affects spore recovery, spore outgrowth, vegetative cell growth and toxin production.

## Methods

### Ethics statement

The collection/use of faecal donations from healthy adult volunteers was approved by the Leeds Institute of Health Sciences and Leeds Institute of Genetics, Health and Therapeutics and Leeds Institute of Molecular Medicine, University of Leeds joint ethics committee (reference HSLTLM/12/061). Written consent was not obtained from participants in order to maintain anonymity, and reduce any embarrassment on the part of the participant. The participation leaflet explained that by providing a sample and giving it to the research team, informed consent for use of the sample in this study was implicit. This consent process was approved by the ethics committee.

### *C*. *difficile* strains

Ten different *C*. *difficile* strains comprising the most common PCR ribotypes in the UK in 2013 (RT015, RT020, RT078, RT023, RT005, RT026, RT027, RT001, RT002, RT014) were selected from those submitted to the *Clostridium difficile* Ribotyping Network (CDRN) during this year. All strains were toxigenic clinical isolates from the UK. Three strains were selected for further studies. RT027 was chosen as an example of this highly epidemic ribotype. RT078 was chosen because strains of this ribotype have also been associated with clinical outbreaks, and are genetically distinct from other ribotypes. RT005 was chosen as an example of a non-epidemic ribotype.

### Preparation of *C*. *difficile* spores

Each strain was reconstituted on Brazier’s Cefoxitin, Cycloserine, Egg Yolk, Lysoyme (CCEYL) agar (final lysozyme concentration 5mg/L). Strains were checked for purity, before single passage on CCEYL agar. After 48 h, growth from each plate was harvested on a sterile swap, and transferred to pre-reduced Colombia Blood Agar (CBA) plates. Plates were incubated anaerobically at 37°C for 14 days, and then all growth was harvested into 50% EtOH in PBS. Spore preps were enumerated on CCEYL agar and visually checked for purity by phase contrast microscopy. Spore preparations were standardised to ~10^7^ cfu/mL.

### Enumeration of *C*. *difficile* spores

Samples were subject to a 10-fold serial dilution series (to 10^−7^) in peptone water. Twenty microliters of each dilution was plated onto Brazier’s CCEYL agar in triplicate and incubated at 37°C. CCEYL agar containing lysozyme was used in order to stimulate germinating spores (unrelated to classical germination pathways) to ensure maximum recovery/enumeration. Colonies were counted from a dilution with ~20–100 colonies to calculate cfu/mL. The limit of detection was 1.22 log_10_ cfu/mL.

### Preparation of faecal filtrate

Faecal samples from 3 healthy, adult volunteers (transported and stored anaerobically) were pooled, mixed with pre-reduced PBS (10%w/v), mixed with a stomacher and passed through a course muslin filter to remove large particulate matter. Aliquots of the resultant slurry were centrifuged (15 mins, 10,000g) and the supernatant sterilised by filtration through 22μm filters. This 10% faecal filtrate (FF) was stored at 4°C for a maximum of 1 week.

### Antibiotic exposure and washing procedure

For each of the ten clinical isolates, replicate spore preps (n = 9) were exposed to 200mg/L fidaxomicin, 200mg/L vancomycin or a non-antimicrobial-containing control in either PBS or FF for 1 hour. Aliquots (1mL) were washed in PBS or FF. During washing, samples were centrifuged (5 minutes, >16,000g), all excess supernatant removed by pipette, and pellets resuspended in wash solution by pipetting. Each aliquot was washed five times, with the sample moved to a fresh Eppendorf tube between washes 3 and 4 to prevent carry over of residual antibiotic. Following washing, spores were frozen at -20°C until subsequent use.

### Determination of active antimicrobial concentration by bioassay

Bioassay agar (100mL) was sterilised by autoclave, cooled to 50°C, seeded with 1mL indicator organism (0.5 MacFarlane standard suspension in PBS) and pored into 245 mm x 245 mm bioassay dishes. Once set, plates were dried, and 25x 9 mm wells dug in the agar (no. 5 cork borer). Washed spore samples (20 μL) were inoculated into wells alongside a doubling dilution calibration series of known antibiotic concentration. After 24 h growth at 37°C, zones of inhibition were measured using callipers accurate to 0.1mm. Calibration lines were plotted from squared zone diameters, and active antimicrobial concentration was calculated from the calibration lines. All samples were assayed in triplicate against a calibration curve of both fidaxomicin and vancomycin. The fidaxomicin bioassay was performed on Wilkins-Charlgren agar with *Kocuria rhizophila* (ATCC 9341) indicator organism, and a calibration series ranging from 2–128 mg/L (limit of detection ~0.5mg/L). The vancomycin bioassay was performed on Muller Hinton Agar with *Staphylococcus aureus* (ATCC 29 213) indicator organism and a 2–128 mg/L calibration series (limit of detection ~1mg/L). Limits of detection refer to the lowest concentration that creates a measurable zone of inhibition. The residual activity of samples following washing was determined using the calibration curve of the relevant exposure antimicrobial.

### Recovery of antibiotic-exposed, washed spores

Following antibiotic exposure and washing in FF, spore aliquots were diluted in a 10-fold series in peptone water to 10^−7^ and enumerated as described above.

### Outgrowth and toxin production following germination of antibiotic-exposed, washed spores

For the three strains investigated in more detail (RT027, RT078, RT005), antibiotic-exposed, FF washed spores were inoculated into pre-reduced Brain Heart Infusion broth (BHI) alongside a non antibiotic-exposed, FF washed control. Samples were taken at 0, 3, 6, 24 and 48 hours post inoculation (triplicate biological and technical replicates for each strain, n = 9). Total viable counts were enumerated on CCEYL agar (as described above) following dilution (10-fold series) in peptone water. Following alcohol shock (50% EtOH for 1 hour), samples were again diluted and inoculated on CCEYL agar to enumerate spores. At each time point, samples (20 μL) were dried on glass microscope slides, and overlaid with Wilkins-Charlgren agar (50 μL) and a glass cover slip for phase microscopy. One hundred entities were recorded from at least three different fields of view, and % phase bright (dormant) spores, phase dark (germinating) spores and vegetative cells calculated. At 6, 24 and 48 h, toxin was enumerated using a cell cytotoxicity assay. Briefly, samples were diluted 10-fold in PBS and applied to a Vero cell monolayer. At 24 and 48 hours, toxin mediated cell rounding was assessed against control wells. Rounding in >80% of cells was considered positive. A sample positive for toxin in the neat well only was assigned a toxin titre of 1 relative unit. A sample positive for toxin in the neat and 10^−1^ wells was assigned a toxin titre of 2 and so on. *Clostridium sordellii* antitoxin was used to confirm specificity.

### Statistical analysis

Statistical significance of differences in viable counts was determined using the two-sample t-test carried out with StataIC 13 software package. P values <0.005 were consisted significant.

## Results

**Fig 1:**
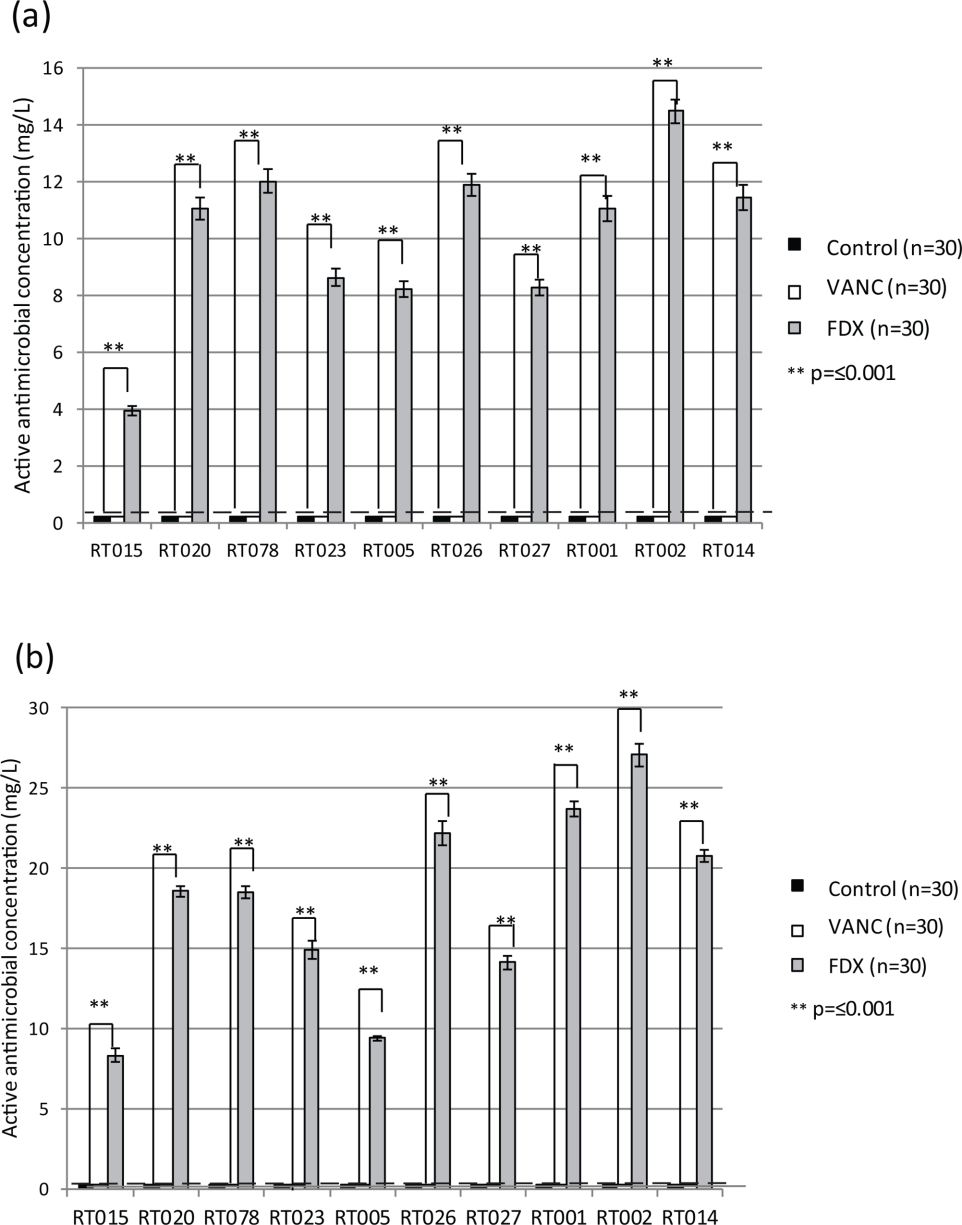
Mean (±SE, n = 9) active antimicrobial concentration post-washing in (a) PBS and (b) FF for spores of 10 different ribotypes. Horizontal dashed line indicated limit of bioassay detection. Statistical significance was determined using two-sample t-test.

**Fig 2:**
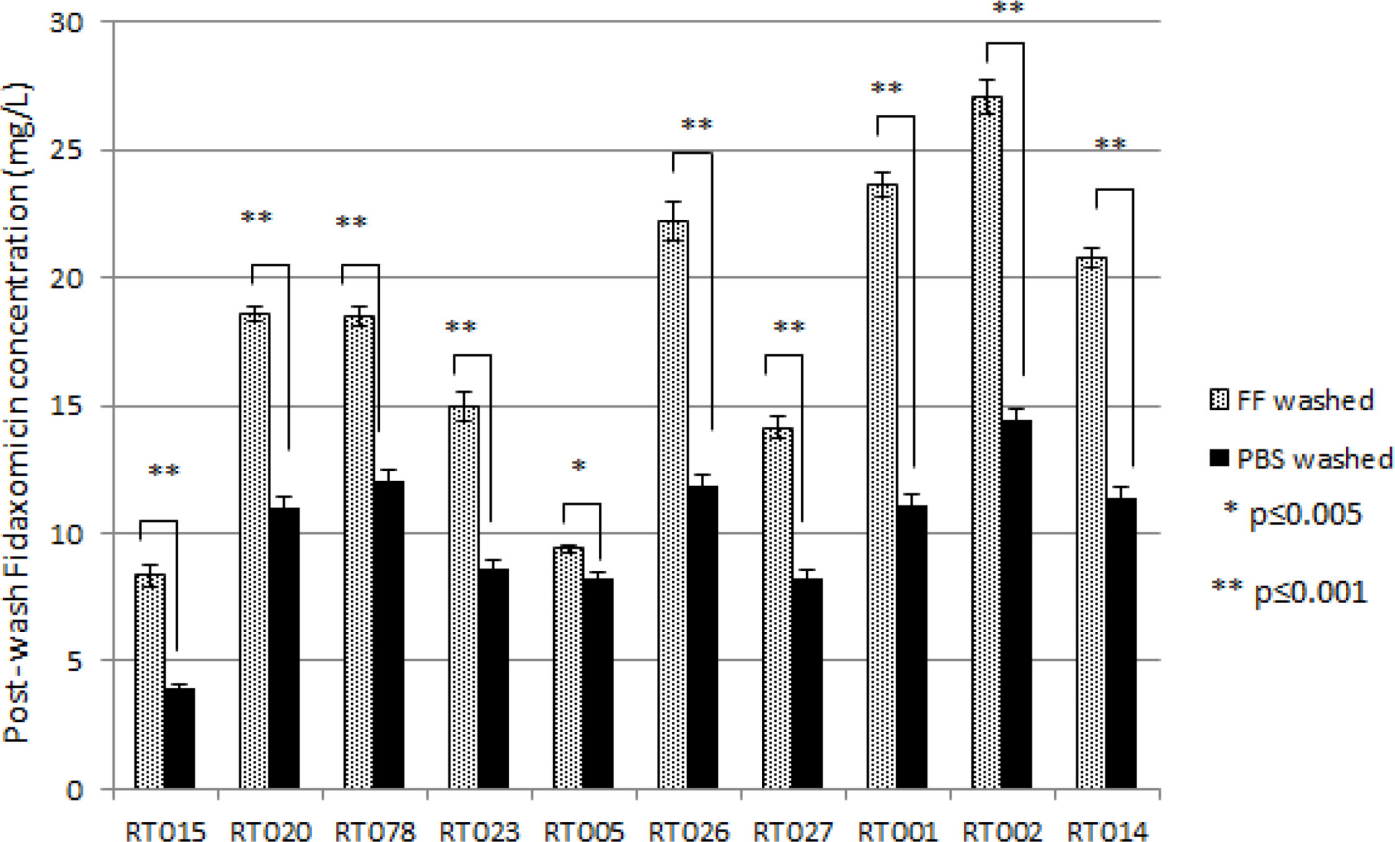
Mean (±SE, n = 9) active fidaxomicin concentration post-washing in PBS or faecal filtrate (FF) for spores of 10 different ribotypes. Statistical significance was determined using two-sample t-test.

Fidaxomicin-exposed spore samples retained detectable antimicrobial activity following washing in PBS, whereas vancomycin-exposed and non-antibiotic-exposed control spore samples did not (Fig 1A). The retention of activity varied between strains and ranged from ~4.0 mg/L (RT015) to ~14.5 mg/L (RT002). The same phenomenon was observed following washing in the more *in vivo* reflective faecal filtrate (FF), with antimicrobial activity again detected in fidaxomicin-exposed, but not vancomycin-exposed or control spores (Fig 1B). Interestingly, washing in FF following fidaxomicin exposure caused significantly greater antimicrobial activity to be retained on spore aliquots compared with PBS (Fig 2). Again this varied according to strain, although the difference was significant (p≤0.005) for all strains. In some cases (RT015, RT026, RT001, RT002), approximately twice the detectable activity was retained following washing in FF compared with PBS (Fig 2).

**Fig 3:**
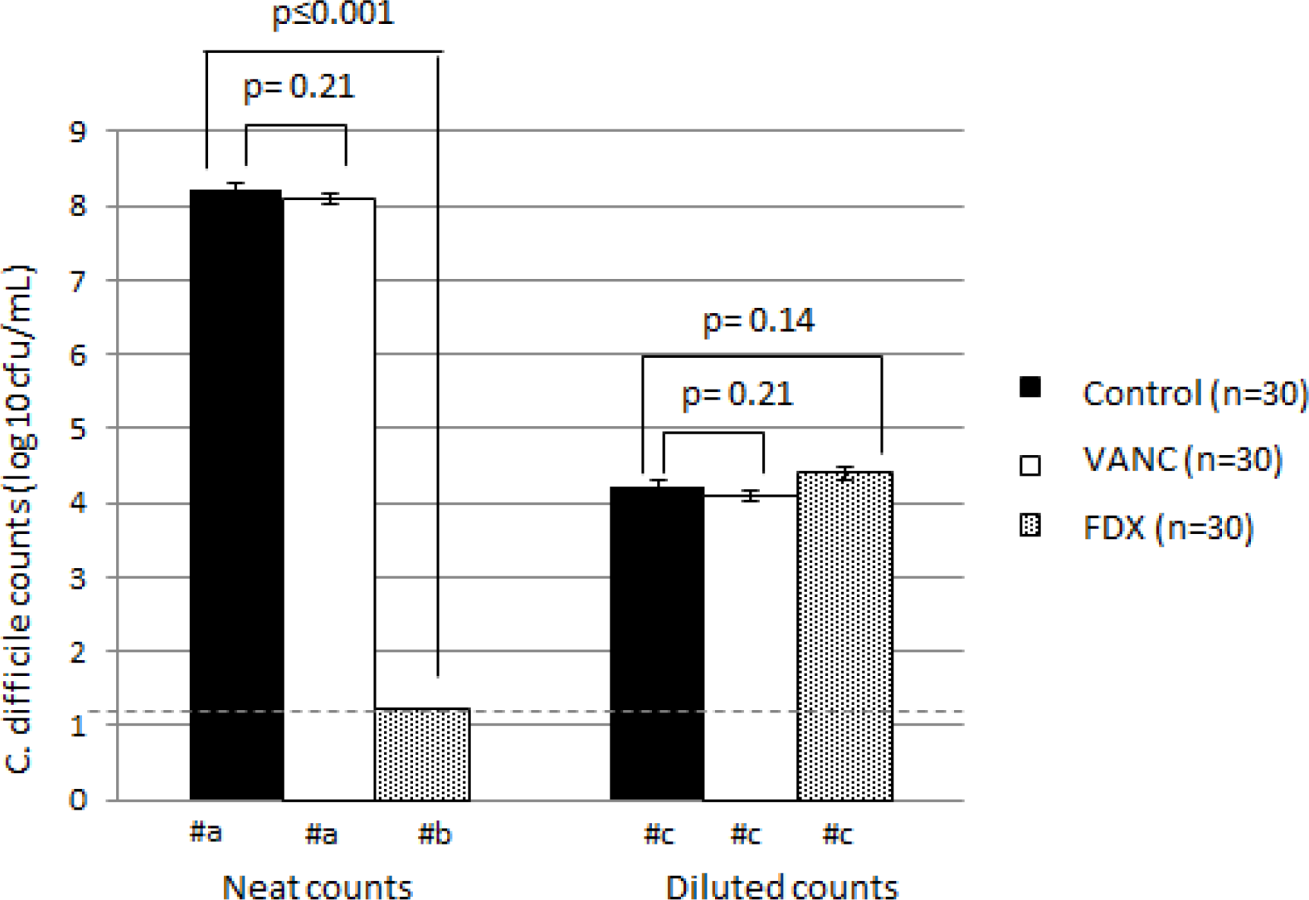
Mean (±SE, n = 30) *C*. *difficile* counts (log_10_ cfu/mL) for all strains following inoculation of faecal filtrate (FF) washed spores onto CCEYL agar. Statistical significance was determined using two-sample t-test. Horizontal dashed line indicates limit of detection (1.22 log_10_ cfu/mL). #a = Too many colonies to count at neat dilution, therefore log_10_ cfu/mL extrapolated from a countable dilution. #b = no growth at neat dilution, log_10_ cfu/mL recorded as limit of detection. #c = log_10_ cfu/mL in 10^−4^ dilution.

Retention of antimicrobial activity prevented recovery of spores on CCEYL agar. No growth was recovered from fidaxomicin-exposed washed spore preps, but recovery from vancomycin-exposed washed spore preps was not significantly different to recovery from control samples (p = 0.21) at ~8 log_10_ cfu/mL (Fig 3). However, this prevention of recovery was lost following dilution of the washed spores. At lower concentrations (≥100-fold dilution), there was no significant difference in the recovery of fidaxomicin-exposed, washed spore preps compared with control samples (p = 0.14) (Fig 3).

**Fig 4:**
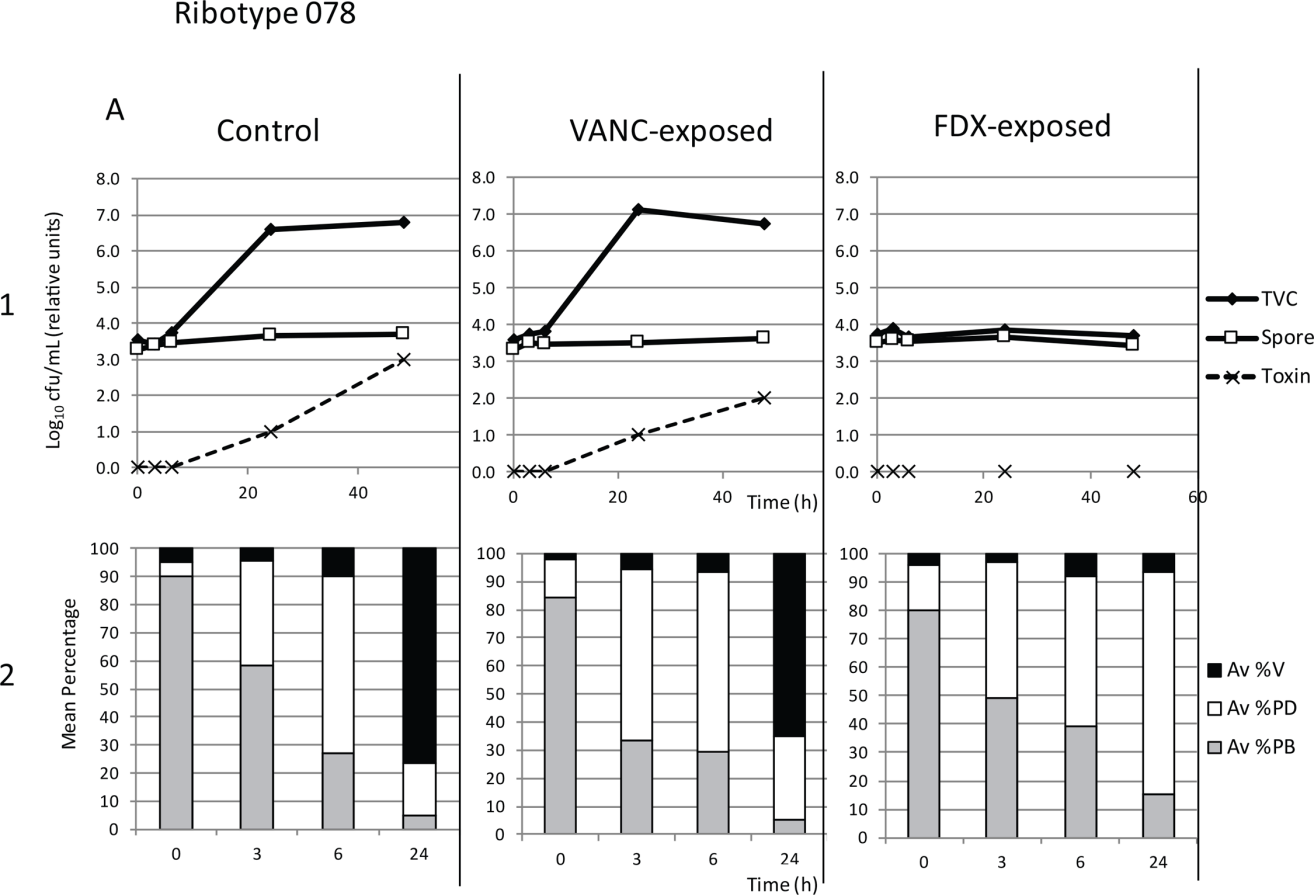
Growth of Control (non-antibiotic exposed washed spores), Vanc-exposed (vancomycin-exposed washed spores) and Fdx-exposed (Fidaxomicin-exposed washed spores) of strain RT078 following inoculation into BHI broth. Panel 1: Mean (n = 9) total viable counts (TVC, log_10_ cfu/mL), spore counts (Spore, log_10_ cfu/mL) and toxin titre (relative units) at tome points 0, 3, 6, 24 and 48 hours. Panel 2: Corresponding mean percentage vegetative cells (Av %V), phase dark spores (Av %PD), and phase bright spores (Av %PB) at time points 0, 3, 6 and 24 hours.

**Fig 5:**
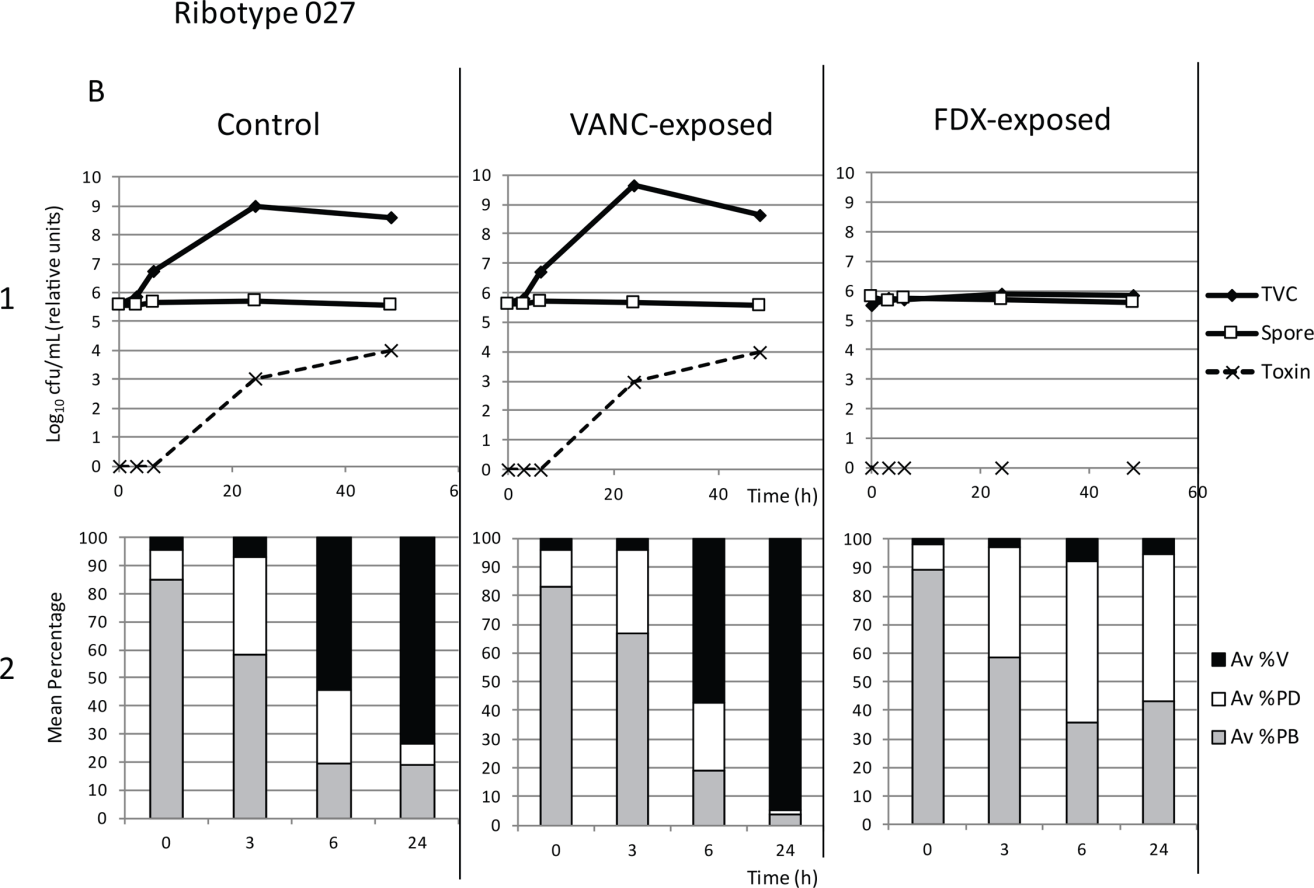
Growth of Control (non-antibiotic exposed washed spores), Vanc-exposed (vancomycin-exposed washed spores) and Fdx-exposed (Fidaxomicin-exposed washed spores) of strain RT027 following inoculation into BHI broth. Panel 1: Mean (n = 9) total viable counts (TVC, log_10_ cfu/mL), spore counts (Spore, log_10_ cfu/mL) and toxin titre (relative units) at tome points 0, 3, 6, 24 and 48 hours. Panel 2: Corresponding mean percentage vegetative cells (Av %V), phase dark spores (Av %PD), and phase bright spores (Av %PB) at time points 0, 3, 6 and 24 hours.

**Fig 6:**
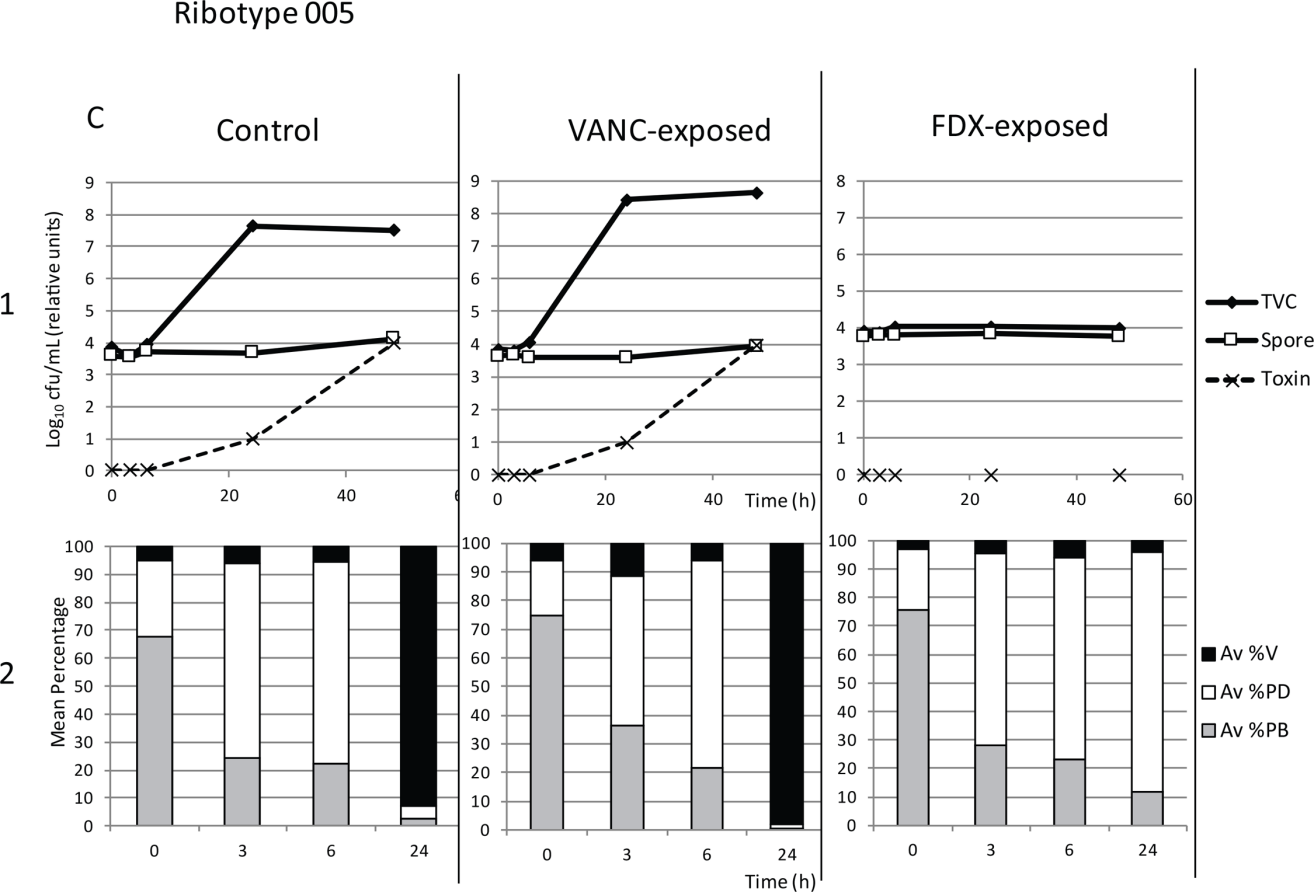
Growth of Control (non-antibiotic exposed washed spores), Vanc-exposed (vancomycin-exposed washed spores) and Fdx-exposed (Fidaxomicin-exposed washed spores) of strain RT005 following inoculation into BHI broth. Panel 1: Mean (n = 9) total viable counts (TVC, log_10_ cfu/mL), spore counts (Spore, log_10_ cfu/mL) and toxin titre (relative units) at tome points 0, 3, 6, 24 and 48 hours. Panel 2: Corresponding mean percentage vegetative cells (Av %V), phase dark spores (Av %PD), and phase bright spores (Av %PB) at time points 0, 3, 6 and 24 hours.

Retention of antimicrobial activity also affected downstream vegetative growth and toxin production of fidaxomicin-exposed washed spores in BHI broth culture, although the early germination events (transformation of phase bright spores to phase dark) were unaffected. In all samples, phase microscopy demonstrated a decrease in percentage phase bright spores and corresponding increase in phase dark spores over the first 3–6 hours as spores germinated (Figs 4–6, panels 2). For each of the three strains assayed (RT027, RT078 and RT005), control (non antibiotic-exposed) washed spores showed an increase in total viable counts compared with spore counts at 24 and 48 h (Figs 4–6). For RT027, this increase was also evident at 6 h (Fig 5). Corresponding phase microscopy confirmed that by 24 hours, the proportion of vegetative cells had increased markedly relative to spores. Vancomycin-exposed washed spores germinated, outgrew and proliferated in the same manner as control samples (Figs 4–6).

No significant differences were observed in the total viable counts of the control and vancomycin-exposed samples at 24 (p = 0.02) or 48 hours (p = 0.11). However, for fidaxomicin-exposed samples, significantly lower total viable counts were observed compared with controls at both 24 and 48 hours (p<0.001). This was confirmed by phase microscopy, where fidaxomicin-exposed spores germinated (decrease in % phase bright spores, increase in % phase dark spores), but did not undergo outgrowth into vegetative cells (%vegetative cells remained lower than control samples, even after 24 h). Importantly, toxin was detected in control and vancomycin-exposed samples after 24 and 48 h, whereas no toxin was identified in fidaxomicin-exposed samples (Figs 4–6).

## Discussion

We have demonstrated that detectable fidaxomicin activity continues to be associated with *C*. *difficile* spores following washing, preventing subsequent spore recovery, whereas vancomycin activity does not. Furthermore, in the three clinical isolates tested here, this persistence of activity prevented vegetative outgrowth and toxin production in batch culture.

It has been widely reported that different *C*. *difficile* strains exhibit variable growth dynamics, sporulation and germination rates and toxin production [17–19]. We have therefore used 10 clinical isolates, belonging to different PCR ribotypes, to evaluate any strain-to-strain variation in the persistence of fidaxomicin activity. This approach helps to strengthen our observations, and ensures observed effects are not strain-specific anomalies. While the extent of fidaxomicin persistence varied between the 10 strains tested here, a detectable active concentration of >4 mg/L was observed in all cases, which is substantially higher than the MIC of fidaxomicin against these 10 strains tested (MIC range = 0.03–0.06 mg/L), and *C*. *difficile* isolates recently reported across Europe (range 0.002–0.25)[20]. It has previously been reported that exposure to both fidaxomicin and vancomycin [21] and also oritavancin [15] inhibits outgrowth of *C*. *difficile* spores, whilst not preventing the early germination events that manifest in the transformation of phase bright spores to phase dark. Therefore, it would be expected that at these supra-MIC levels, persisting fidaxomicin activity would act on germinating spores, preventing outgrowth (and hence recovery on agar). This is confirmed by the data presented here showing that fidaxomicin-exposed spores could not be recovered on CCEYL agar, whereas control and vancomycin-exposed spores were recovered.

Following inoculation into BHI, non-antimicrobial exposed control spores and vancomycin exposed spores germinated, and outgrew into a proliferating vegetative population, despite no additional supplementation with classical germinants. Other authors have also reported germination of clinical isolates in the absence of additional supplementation with classical germinants [17]. BHI was used as a toxin production promoting media to assess downstream affects on toxin levels. Fidaxomicin-exposed spores inoculated into BHI undergo early germination events (as shown by transformation of spores from phase bright to phase dark), but no outgrowth to vegetative cells occurred. This is in agreement with previously published work. [21] Crucially from a clinical standpoint, this lack of vegetative cell growth led to a lack of detectable toxin–the key disease determinant in CDI.

The mechanism for fidaxomicin association with spores remains unknown, but the antibiotic has also been shown to adhere to glassware. [22] It is likely that fidaxomicin adheres to the exosporium of *C*. *difficile* (as has recently been shown for ramoplanin[16]), potentially due to electrostatic charges resulting form cross-linkages on the spore surfaces. The presence of the exosporium has been shown to increase hydrophobicity of *C*. *difficile* spores, and affect adherence to cells[23]. Fidaxomicin is a hydrophobic molecule, and it is therefore probable that spore hydrophobicity is an important factor in its adherence; strain-to-strain differences in spore exosporium and hydrophobicity may contribute to the observed variation in levels of fidaxomicin persistence. It would be desirable to directly visualise the binding of fidaxomicin to spores; however, conjugation of fluorescent markers to fidaxomicin is problematic due to the lack of amenable functional groups. Future experiments aim to explore alternative techniques for imaging of fidaxomicin such as Imaging Mass Spectrometry.

Kraus et al [16] demonstrated that ramoplanin binds to the exosporium of *C*. *difficile*, preventing recovery on agar plates. Interestingly, they also reported that this effect can be diluted out, and postulate that a concentration equilibrium is achieved between the exosporium and the supernatant over time; dilution lowers the local antimicrobial concentration to below the MIC, thereby allowing spore outgrowth. Our data suggest that the same dilution effect occurs with fidaxomicin, and once the local concentration on the plate falls below the MIC (1:100 dilution required for fidaxomicin-exposed spores to grow, whereas 1:10 dilution allowed growth of ramoplanin-exposed spores [16]), spore recovery returns to normal. This hypothesis would also explain the results following inoculation into BHI. If a concentration equilibrium is achieved between the spores and supernatant, this would be likely to be a supra-MIC concentration (thereby preventing spore outgrowth and vegetative proliferation, hence preventing toxin production). However, the dilution process associated with enumeration then lowers the concentration enough to allow recovery on CCEYL.

Persisting fidaxomicin activity can be removed by washing spores in ethanol or DMSO [24], which is likely to be due to the high solubility of fidaxomicin in these solvents. However, investigating persistence of activity following washing of spores in PBS, ethanol or DMSO bears little resemblance to conditions in the colon, and is likely to have little significance in elucidating the importance of this phenomenon clinically. We have therefore investigated the persistence of activity following washing in faecal filtrate. Whilst this is still not fully representative of the complex, multispecies environment of the colon, it is far more clinically reflective than other washing agents. Interestingly, when spores were washed in faecal filtrate, the level of detectable fidaxomicin activity was greater than following washing in PBS. The reasons for this are unclear, but are likely to relate to the biophysical properties of faeces, e.g. salt concentrations affecting solubility. Taken with the fact that persistence of activity following fidaxomicin treatment and a lack of spore recovery have both been reported in a clinically reflective human gut model,[7] this suggests that this persistence of activity on spores is likely to occur *in vivo*.

The persistence of fidaxomicin activity on spores may confer an advantage over other CDI treatment agents such as vancomycin. The data presented here show that in batch culture this persistence prevents downstream outgrowth of spores into vegetative cells, and crucially subsequent toxin production, suggesting that this is likely to be important factor in the observed reduced rates of recurrence following fidaxomicin treatment. Recurrence is a major issue in CDI treatment, with ~25% of patients experiencing a re-occurrence of symptoms following treatment. Recurrence can be due to a recrudescence of spores remaining in the gut following an infection, or a re-infection of a still-susceptible patient with a ‘new’ infective dose of *C*. *difficile*.[3] Comparative trials have reported that fidaxomicin is superior to vancomycin in prevention of recurrences, particularly in the first two weeks after antibiotic cessation.[8] We have previously postulated that this may be due to a persistence of fidaxomicin in the gut as observed in an *in vitro* gut model [7] and in patients during Phase I human volunteer studies.[25] However, specific persistence on any residual spores remaining in a patient’s gut following fidaxomicin treatment may have additional benefits. This would localise the persisting activity to the germinating spore, where it can act early to prevent outgrowth, therefore preventing recrudescence of vegetative cell proliferation and toxin production. To this end, dosing strategies that maximise persistence of fidaxomcin activity in the gut are being investigated. [11, 26] If detectable activity can persist on spores ‘shed’ by fidaxomicin treated patients, this may have important infection control benefits in helping to prevent cell growth following spore transmission. The level of persistence on spores in the environment, and the clinical effects of this persistence should be investigated further. It would also be of interest to investigate the longevity of persisting fidaxomicin activity on *C*. *difficile* spores, and whether this persistence affects the ability of spores to initiate an infection in an *in vitro* gut model, or when transferred orally in a rodent model of CDI.
